# A Rare Case of Streptococcus agalactiae Ventriculitis and Endocarditis

**DOI:** 10.7759/cureus.56151

**Published:** 2024-03-14

**Authors:** Ozioma Akahara, Robert Hennis, Jared J Bies, Aymara Y Chang

**Affiliations:** 1 Internal Medicine, Texas Tech University Health Sciences Center Paul L. Foster School of Medicine, El Paso, USA

**Keywords:** infective endocarditis cerebral infarction, complicated infective endocarditis, valvular endocarditis, infective endocarditis complications, infective endocarditis, infective strep. agalactiae endocarditis, ventriculitis, brudzinski sign ventriculitis, bacterial meningitis, strep. agalactiae

## Abstract

*Streptococcus agalactiae *infection is typically seen in specific populations, including neonates, pregnant women, and the elderly. These patients have immature, lower, and waning immune systems, which makes them more susceptible to infections. Typical *S. agalactiae* infections manifest as cellulitis, bacteremia, endocarditis, meningitis, ventriculitis (a rare complication of meningitis), and osteomyelitis. In rare cases, a patient can present with two or more of these typical infection manifestations. The authors present a case of a 48-year-old female with a past medical history of hypothyroidism and chronic back pain who presented to the emergency department with altered mental status. The patient developed nausea and vomiting two days prior to presentation after a family gathering, followed by occipital headache and agitation. On arrival at the emergency department, the patient did not follow commands and was drowsy. The initial examination showed positive for Brudzinski and Kernig signs. The patient was tachycardic, tachypneic, and hypertensive. Initial computed tomography (CT) head without contrast was negative for any acute pathology. Neurology was consulted, and a bedside lumbar puncture was performed, which was significant for elevated opening pressure of 32 cm H_2_O.

The patient was initially started on ceftriaxone, ampicillin, vancomycin, acyclovir, and dexamethasone. Magnetic resonance imaging (MRI) of the brain with and without contrast showed acute ventriculitis, mild leptomeningeal enhancement, and a right posterior corona radiata acute lacunar infarct. Meningitis panel, BioFire (BioFire Diagnostics, Salt Lake City, UT), was positive for *S. agalactiae*, and the patient was de-escalated to ceftriaxone*. *Cerebrospinal fluid and blood cultures returned positive for *S. agalactiae*. A transthoracic echocardiogram was negative for endocarditis, but a transesophageal echocardiogram was significant for a 0.7 × 0.4 cm mobile echodensity attached to the posterior leaflet of the mitral valve (P1/P2 scallop). Repeat blood cultures, additional cerebrospinal fluid analysis, and infectious workup remained negative. Cardiology was consulted and recommended medical treatment. The patient improved clinically, continued ceftriaxone, and was discharged to complete a total of six weeks of treatment with outpatient follow-up evaluations. This case depicts a rare presentation of endocarditis, meningitis, and ventriculitis *S. agalactiae *infection and the need for a definite treatment algorithm in the management of complicated conditions such as the one presented.

## Introduction

The *Streptococcus* genus are gram-positive pathogens with spherical or ovoid cells that characteristically take on the chain formation and occasionally are arranged in pairs [1]. This genus is known for its Lancefield classification, ranging from groups A to M, based on their cell wall characteristics and antigenic reactions [1,2]. They can be further classified into a pyogenic group, consisting of *Streptococcus agalactiae* and *Streptococcus pyogenes*, and a mitis group, including *Streptococcus pneumoniae*, *Streptococcus mitis*, and *Streptococcus oralis* [2]. Their infectivity relies on their cell-surface components and virulence factors, which comprise biofilm, adhesins, capsules, M-protein, and many more characteristics [1].

*S. agalactiae* made its first presentation in literature in the 1930s when Lancefield differentiated it from other pathogens in its genus after it had been isolated from cow’s milk and bovine mastitis [2,3]. Now, it is commonly known as group B streptococci (GBS), based on its Lancefield classification. Infection by *S. agalactiae* presents as cellulitis, bacteremia, endocarditis, meningitis, ventriculitis (a rare complication of meningitis), and osteomyelitis [3]. Its infectivity is associated with specific populations, particularly pregnant women and infants [3]. However, there is evidence of increasing incidence of *S. agalactiae* infection in the adult non-pregnant population, evidenced by data accrued from the Active Bacterial Core surveillance that showed an increase from 8.1 cases per 100,000 in 2008 to 10.9 cases per 100,000 in 2016 [3,4]. Non-pregnant adults who are infected by *S. agalactiae* often have other comorbidities or are immunocompromised or older [3]. In this report, the authors present a patient with ventriculitis and endocarditis *S. agalactiae* infection.

## Case presentation

A 48-year-old female with a past medical history of hypothyroidism and chronic back pain presented to the emergency department (ED) with altered mental status. On presentation to the ED, the patient was not following commands and was drowsy. Per the patient’s husband, the patient had suffered from food poisoning after a family gathering and experienced nausea, vomiting, and gastrointestinal upset two days prior. The patient also developed a constant occipital headache.

Initial vital signs were significant for a temperature of 37.0°C, tachycardia with a heart rate of 116, tachypnea at 23 breaths per minute, hypotension of 99/54 with a mean arterial pressure of 69, and an oxygen saturation of 97% on room air. On initial examination, the patient could not follow commands, was drowsy, and had a Glasgow coma scale score of 10. The patient had positive Brudzinski and Kernig signs and no focal neurological deficits. Oral examination showed poor dentition. Initial laboratory findings showed a normal white blood cell count with a bandemia, mild non-anion gap metabolic acidosis, uncontrolled hypothyroidism, hypoalbuminemia, hypoproteinemia, transaminitis, elevated alkaline phosphatase, and an elevated creatinine kinase (Table 1).

**Table 1:** Initial laboratory workup

Serum test	Normal range	Result
White blood cells	4.50-11.00 × 10^3^/μL	7.03 × 10^3^/μL
Bands	0.00-1.21 × 10^3^/μL	2.53 × 10^3^/μL
Hemoglobin	12.0-15.0 g/dL	11.3 g/dL
Hematocrit	36.0-47.0%	32.4%
Mean corpuscular volume	82.0-98.0	93.6
Platelets	150-450 × 10^3^/μL	96 × 10^3^/μL
Sodium	135-145 mmol/L	137 mmol/L
Potassium	3.5-5.1 mmol/L	4.1 mmol/L
Chloride	98-107 mmol/L	106 mmol/L
Bicarbonate	22-30 mmol/L	20 mmol/L
Glucose	74-106 mg/dL	97 mg/dL
Blood urea nitrogen	7-17 mg/dL	11 mg/dL
Creatinine	0.52-1.04 mg/dL	0.70 mg/dL
Calcium	8.4-10.2 mg/dL	8.3 mg/dL
Albumin	3.5-5.0 g/dL	2.9 g/dL
Total protein	6.3-8.2 g/dL	5.2 g/dL
Serum osmolality	281-303 mOsm/kg	283 mOsm/kg
Aspartate transaminase	14-36 IU/L	69 IU/L
Alanine transaminase	0-35 IU/L	61 IU/L
Alkaline phosphate	38-126 IU/L	207 IU/L
Creatinine kinase	30-135 IU/L	493 IU/L
Thyroid-stimulating hormone	0.465-4.680 mIU/L	60.900 mIU/L
Troponin	0.000-0.034 ng/mL	<0.012 ng/mL
Ammonia	9.0-30.0 μmol/L	10.8 μmol/L

**Figure 1:**
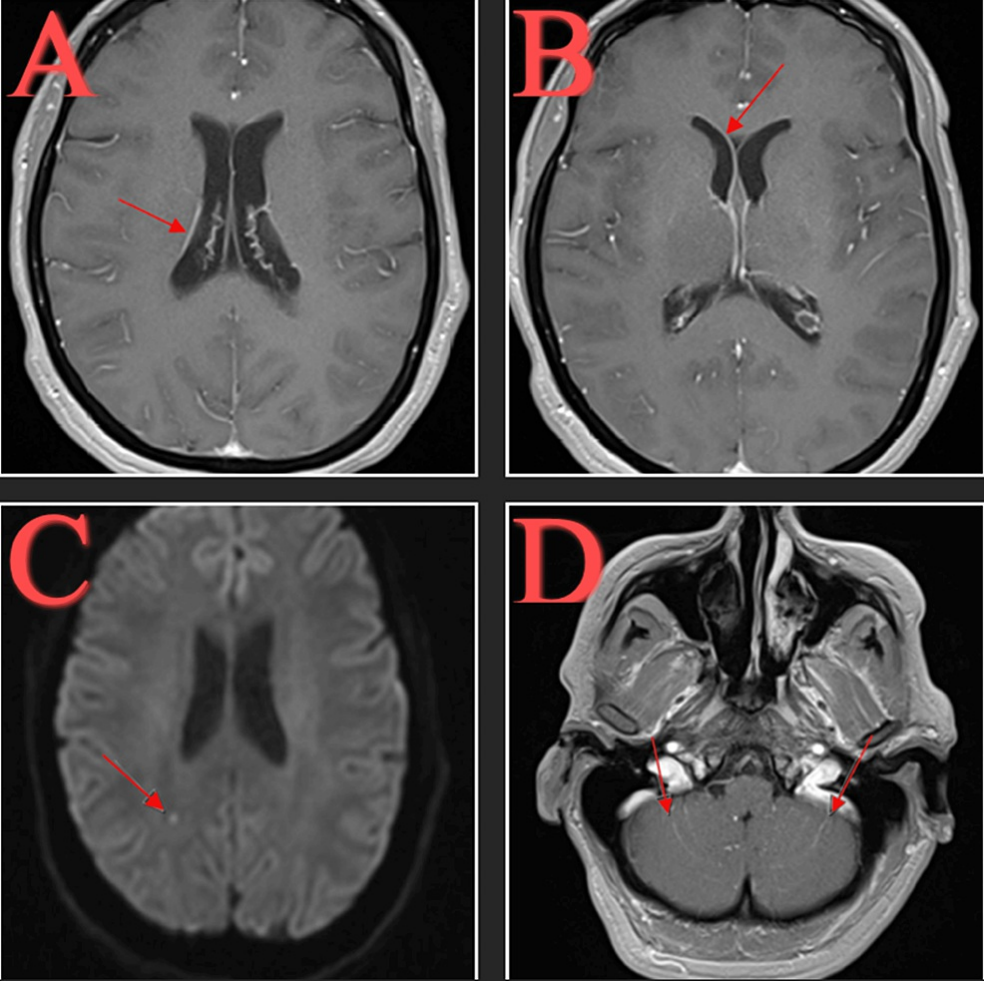
MRI brain with and without contrast. Mild enhancement of the ependymal lining of the ventricles (concerning ventriculitis) (A,B). Right posterior corona radiata acute lacunar infarct (C). Mild enhancement of the folia of the cerebellar hemispheres (D).

An electrocardiogram (EKG) showed sinus tachycardia, incomplete bundle branch block, and a nonspecific T wave abnormality. An initial computed tomography (CT) head without contrast showed no acute intracranial abnormality. The patient had a bedside lumbar puncture, which showed an elevated opening pressure of 32 cm H_2_O. The patient was started on empiric intravenous ceftriaxone, ampicillin, vancomycin, acyclovir, and dexamethasone. The patient had a magnetic resonance imaging (MRI) brain with and without contrast for suspected meningitis that was significant for minimal debris layering in the bilateral occipital horns and mild enhancement of the ependymal lining of the ventricles (concerning ventriculitis), mild enhancement of leptomeningeal area postcontrast and the folia of the cerebellar hemispheres, and a right posterior corona radiata acute lacunar infarct (Figure 1).

Cerebrospinal fluid (CSF) analysis showed neutrophilic pleocytosis, hyperproteinorrhachia, elevated red blood cells, and normal glucose (Table 2). Meningitis panel, BioFire (BioFire Diagnostics, Salt Lake City, UT), was positive for *S. agalactiae*.

**Table 2:** Cerebrospinal fluid analysis

Test	Reference range	Result
Opening pressure	10-20 cm H_2_O	32 cm H_2_O
Color	Colorless	Colorless
Appearance	Clear	Hazy
Red blood cells	<5 μL	40 μL
White blood cells	<5 μL	1,263 μL
Glucose	40-70 mg/dL	43 mg/dL
Protein	12-60 mg/dL	268 mg/dL
Streptococcus agalactiae	Negative	Positive

Electroencephalography (EEG) showed a diffuse slow background with sleep spindles, and there were fewer fast frequencies on the right hemisphere during stimulation, suggestive of moderate diffuse encephalopathy with a greater degree of right hemispheric neuronal dysfunction with no epileptiform abnormalities or electroclinical seizures recorded. Repeat CT head without contrast was significant for mild dental disease. An MRI of the cervical, thoracic, and lumbar spine with and without contrast was done to further characterize the degree of meningitis and showed subtle leptomeningeal enhancement along the upper cervical cord, along the cerebellar folia, and at the caudal terminus of the thecal sac. Initial blood cultures from admission were obtained and were positive for gram-positive cocci in clusters, and susceptibilities showed multi-susceptible *S. agalactiae* (Table 3).

**Table 3:** Streptococcus agalactiae susceptibilities I, intermediate; MIC, minimal inhibitory concentration; R, resistant; S, sensitive

Drug	*Streptococcus agalactiae* (group B)	
	Interpretation	MIC
Amoxicillin	S	≤0.25
Cefotaxime	S	≤0.0625
Ceftriaxone	S	≤0.0625
Clindamycin	I	0.5
Erythromycin	R	4.0
Levofloxacin	S	≤0.5
Linezolid	S	≤0.5
Penicillin	S	≤0.03125
Vancomycin	S	<0.25

Infectious disease was consulted, and a transthoracic echocardiogram, along with a high dose ceftriaxone for a minimum of two weeks if no vegetations, and for six weeks if vegetations were present, were recommended. Transthoracic echocardiogram showed normal left ventricular systolic function, with an ejection fraction estimate of 55-60%, mild mitral regurgitation, no pericardial effusion, and no echocardiographic evidence of endocarditis.

**Figure 2:**
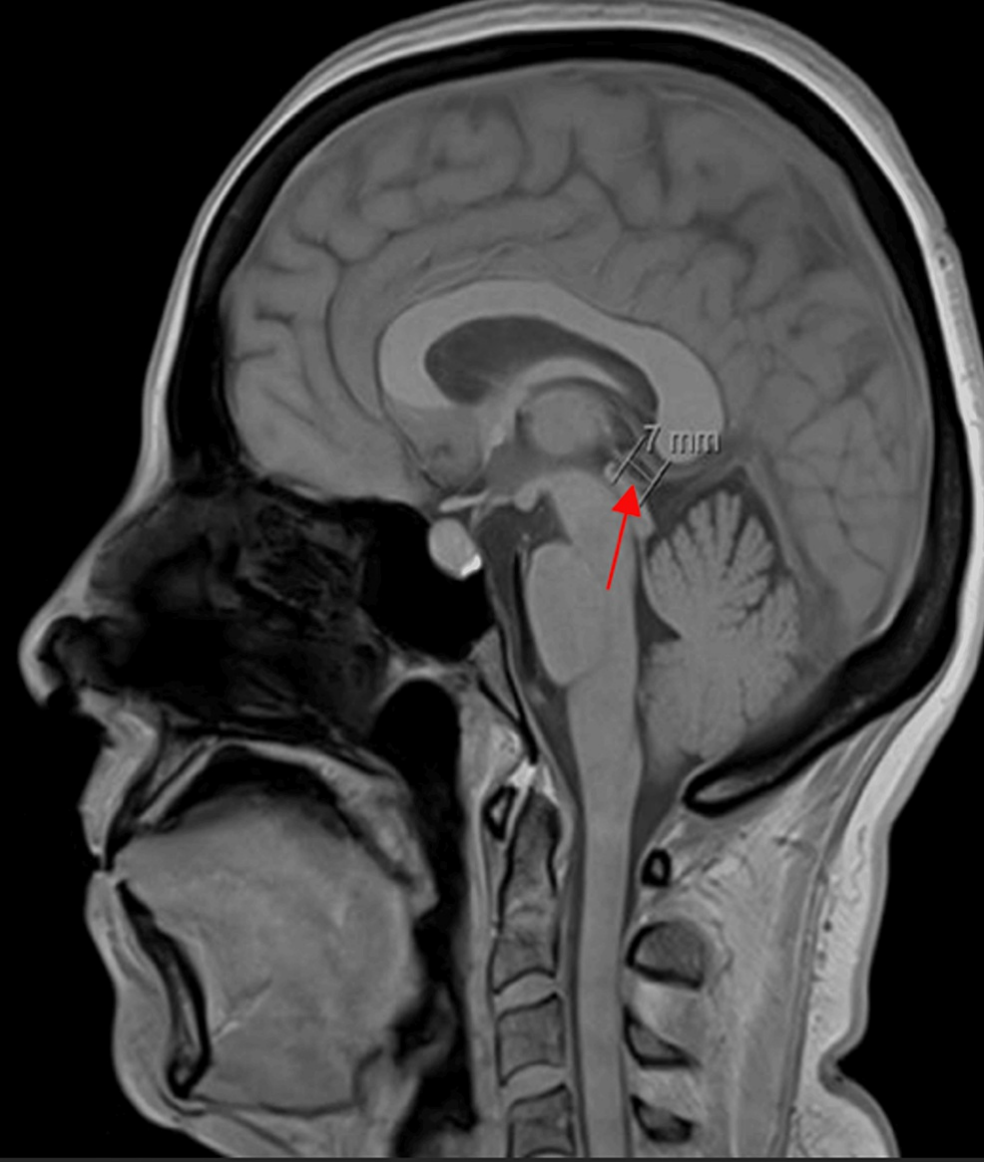
A 7-cm pineal gland cyst.

During admission, the patient complained of continued throbbing pain radiating from the head to her eyes and shoulder. A repeat MRI brain with and without contrast due to continued headaches showed progression of ventriculitis, interval increase in size of areas of restricted diffusion within debris within bilateral posterior horns of lateral ventricles with enhancement involving ependymal linings, supratentorial and infratentorial leptomeningitis, and a 7-mm pineal gland cyst (Figure 2).

**Figure 3:**
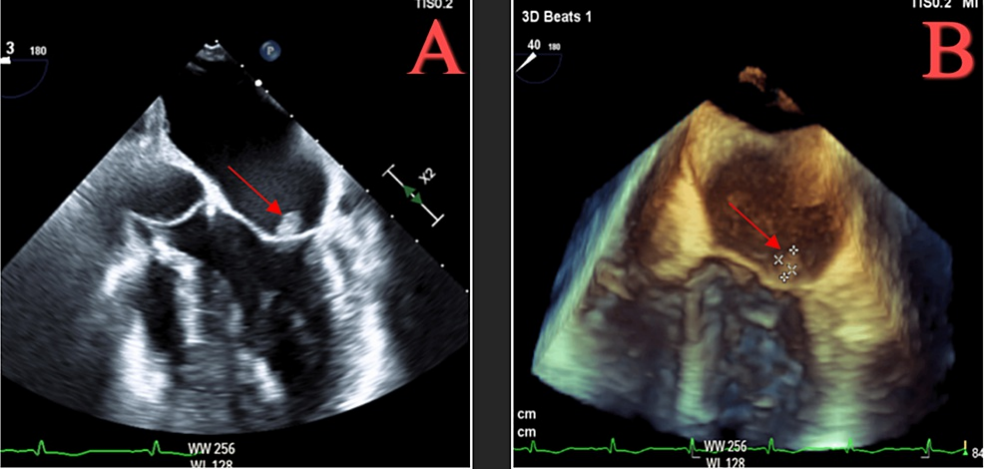
Transesophageal echocardiogram showing 0.7 × 0.4 cm mobile echodensity attached to the posterior leaflet of the mitral valve (A,B).

The imaging was reviewed by a neurologist, who recommended increasing the topiramate dose as needed for headaches. The patient’s headache improved with the addition of topiramate. Given high suspicion for endocarditis with associated meningitis/ventriculitis due to *S. agalactiae*, a transesophageal echocardiogram (TEE) was completed and was significant for a 0.7 × 0.4 cm mobile echodensity attached to the posterior leaflet of the mitral valve (P1/P2 scallop) (Figure 3).

**Figure 4:**
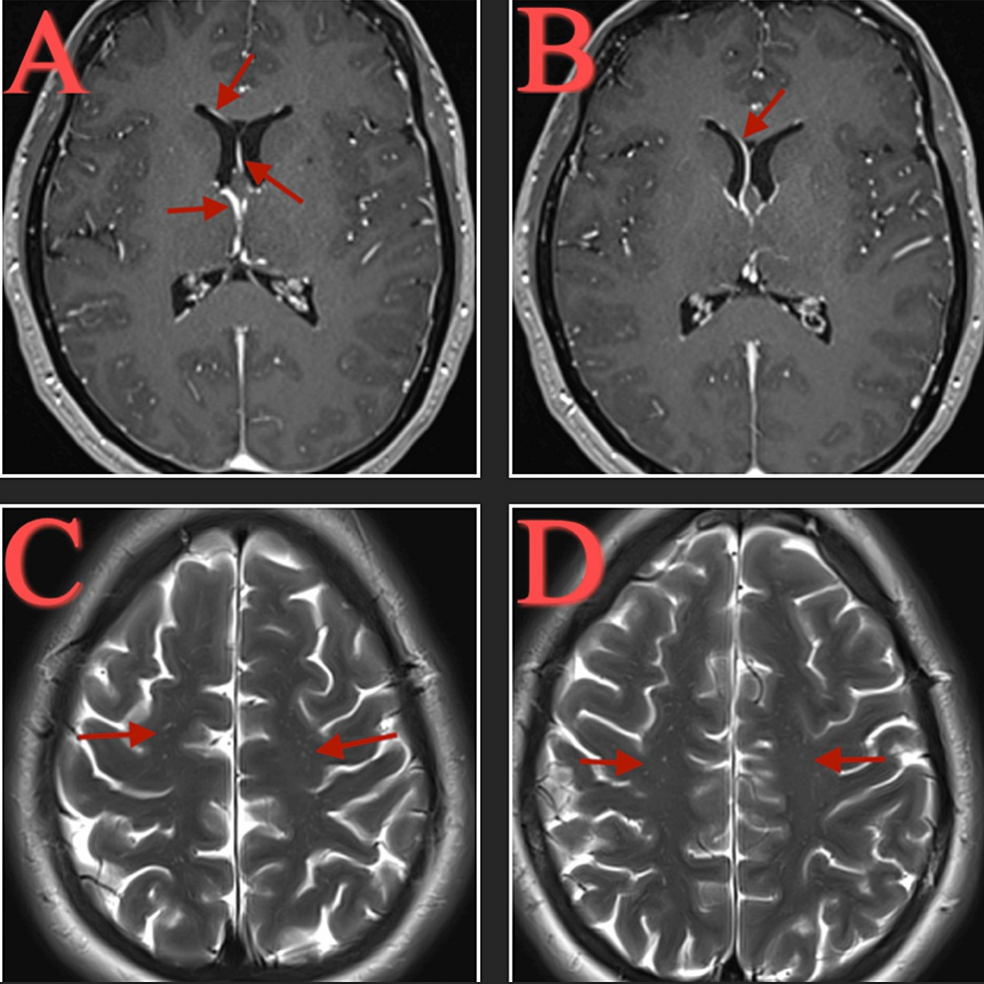
MRI brain with and without contrast. Persistent enhancement of ependymal linings signifying ventriculitis (A,B). Increased conspicuity of punctate foci of enhancement in the frontal and parietal corona radiata, corresponding to evolving tiny subacute infarcts (C,D).

Cardiology was consulted for native valve endocarditis and recommended no surgical interventions. Repeat MRI of the brain with and without contrast showed interval decrease in the amount of layering debris in the lateral ventricles with persistent enhancement involving the ependymal linings, decreased leptomeningeal enhancement, with increased conspicuity of punctate foci of enhancement in the right frontal and parietal corona radiata, corresponding to evolving tiny subacute infarcts (Figure 4).

The patient had a peripherally inserted central catheter (PICC) inserted and was instructed to complete intravenous (IV) ceftriaxone for a total of six weeks from negative blood cultures. The patient was discharged home with home health with scheduled follow-ups with a primary care provider, neurology, and cardiology in an outpatient setting.

## Discussion

*S. agalactiae* is a gram-positive pathogen known popularly by its Lancefield classification as GBS [1]. GBS infection is increasingly common within specific populations: pregnant women, women in the peri-partum stage, and neonates [5]. However, there has been a noted growth in incidence in populations outside of the aforementioned, such as among individuals greater than the age of 60, individuals with diabetes mellitus, and individuals in an immunocompromised state [6]. Clinical presentation of GBS infection includes infective endocarditis, pneumonia, skin infections, osteomyelitis, and more [6].

GBS meningitis in adults remains uncommon, and patients with GBS meningitis with coinciding endocarditis are especially rare. In the exhaustive literature review conducted by van Kassel et al., only 14 patients were found with both conditions [7]. Yet rates of GBS infection in adults are rising globally [4]. The disease’s low incidence, high mortality rate, and potential for an increasing incidence in future years highlight the importance of this case.

Treatment algorithms for GBS meningitis with endocarditis are not fully standardized, and the disease course of the patient, in this case, can help future clinicians and researchers develop an evidence-based standard of care. In the van Kassel study, antimicrobial treatment was detailed in 111 of the 144 patients, with 67 receiving penicillin G (either in monotherapy or combined with gentamicin (n = 9) or vancomycin (n = 1)) and 14 receiving ceftriaxone monotherapy [7]. The Infectious Disease Society of America (IDSA) indicates that penicillin G and ampicillin are first-line, associated with A-III-level evidence, and ceftriaxone as an alternative, associated with B-III evidence [8]. However, these guidelines have not been updated since 2004, and there is some evidence of increasing antibiotic resistance to penicillin G in GBS in some areas [9]. Additionally, sometimes, penicillin is not practical for home use since it requires more frequent administration than ceftriaxone. Antibiotic stewardship considerations favor penicillin. Given the rarity of adult GBS meningitis/endocarditis, more studies are needed to determine the most effective treatment, but it is notable that ceftriaxone was associated with a generally favorable outcome in this patient.

This case underscores the importance of evaluating GBS meningitis patients for endocarditis and other associated conditions and sequelae. In the van Kassel study, concomitant endocarditis was noted in GBS meningitis patients at a much higher level (12%) than in meningitis patients generally (1%) and raised the lethality of GBS meningitis patients from 31% to 36% [7,10]. Although rare, the risk of having GBS meningitis with concomitant endocarditis is significant enough for clinicians to have a high suspicion for it and work it up appropriately [11]. Other studies have documented substantial sequelae resulting from the additional endocarditis burden in patients with meningitis, including respiratory failure, circulatory shock, and arthritis [10]. Perhaps most importantly, the presence of endocarditis typically extends antibiotic treatment for meningitis from two weeks to six weeks [10]. Recommendations to screen for endocarditis in patients with *Staphylococcus aureus* meningitis, group D *Streptococcus* meningitis, and enterococcal meningitis have already been published [12]. Given the increase in morbidity and mortality in GBS meningitis with concomitant endocarditis, as well as divergent treatment requirements when endocarditis is present, we agree that all patients with GBS meningitis should be screened for endocarditis.

This patient’s hospital course proceeded with multiple noteworthy events. First, the patient was noted to have an acute right posterior corona radiata lacunar infarct on brain MRI. Bacterial meningitis is known to increase the risk of many types of CNS sequelae, infarction among them [13]. Another notable factor in the patient’s disease course was her significant hypothyroidism. Hypothyroidism has immunosuppressive effects thought to predispose to infection in general, and one report implicates hypothyroidism in a patient’s suboptimal immune response to meningitis specifically [14,15].

## Conclusions

GBS meningitis with concurrent endocarditis is a rare and highly lethal illness. Epidemiological trends suggest it may be becoming more prevalent, which increases the relevance of this case presentation. This case can help support the refinement of treatment algorithms for GBS meningitis/endocarditis and serve as a reminder for physicians to consider screening for endocarditis in all GBS meningitis cases for appropriate, timely diagnosis and treatment.
